# Application of the Team Emergency Assessment Measure for Prehospital Cardiopulmonary Resuscitation

**DOI:** 10.3390/jcm11185390

**Published:** 2022-09-14

**Authors:** Sangsoo Han, Hye Ji Park, Won Jung Jeong, Gi Woon Kim, Han Joo Choi, Hyung Jun Moon, Kyoungmi Lee, Hyuk Joong Choi, Yong Jin Park, Jin Seong Cho, Choung Ah Lee

**Affiliations:** 1 Department of Emergency Medicine, Soonchunhyang University Bucheon Hospital, Bucheon 14584, Korea; 2 Department of Emergency Medicine, Hallym University, Dongtan Sacred Heart Hospital, Hwaseong 18450, Korea; 3 Department of Emergency Medicine, Catholic University of Korea, St. Vincent’s Hospital, Seoul 06591, Korea; 4 Department of Emergency Medicine, Dankook University Hospital, Cheonan 31116, Korea; 5 Department of Emergency Medicine, College of Medicine, Soonchunhyang University, Cheonan 31151, Korea; 6 Department of Emergency Medicine, Myongji Hospital, Goyang 10475, Korea; 7 Department of Emergency Medicine, Hanyang University Guri Hospital, Guri 11923, Korea; 8 Department of Emergency Medicine, Chosun University Hospital, Gwangju 61453, Korea; 9 Department of Emergency Medicine, Gil Medical Center, Gachon University College of Medicine, Incheon 21565, Korea

**Keywords:** Team Emergency Assessment Measure (TEAM), prehospital cardiopulmonary resuscitation (CPR), out-of-hospital cardiac arrest (OHCA)

## Abstract

Introduction: Communication and teamwork are critical for ensuring patient safety, particularly during prehospital cardiopulmonary resuscitation (CPR). The Team Emergency Assessment Measure (TEAM) is a tool applicable to such situations. This study aimed to validate the TEAM efficiency as a suitable tool even in prehospital CPR. Methods: A multi-centric observational study was conducted using the data of all non-traumatic out-of-hospital cardiac arrest patients aged over 18 years who were treated using video communication-based medical direction in 2018. From the extracted data of 1494 eligible patients, 67 sample cases were randomly selected. Two experienced raters were assigned to each case. Each rater reviewed 13 or 14 videos and scored the TEAM items for each field cardiopulmonary resuscitation performance. The internal consistency, concurrent validity, and inter-rater reliability were measured. Results: The TEAM showed high reliability with a Cronbach’s alpha value of 0.939, with a mean interitem correlation of 0.584. The mean item–total correlation was 0.789, indicating significant associations. The mean correlation coefficient between each item and the global score range was 0.682, indicating good concurrent validity. The mean intra-class correlation coefficient was 0.804, indicating excellent agreement. Discussion: The TEAM can be a valid and reliable tool to evaluate the non-technical skills of a team of paramedics performing CPR.

## 1. Introduction

Teamwork and leadership are known to be important non-technical skills (NTSs) in healthcare [1]. NTS are defined as “the cognitive, social, and personal resource skills that complement technical skills and contribute to safe and efficient task performance” [2]. NTSs include situational awareness, decision making, communication, teamwork, leadership, stress management, and coping with fatigue [3]. These are important for preventing medical errors and ensuring patient safety [4]. Moreover, in many studies, NTSs were correlated with the high clinical performance of the team. 

Teamwork plays an important role in the prevention of adverse events [5]. A large-scale survey by the UK National Health Service reported that efficient teams had lower levels of patient mortality and sickness absence [6]. In another study, the implementation of a team training program reduced the surgical mortality rate of inpatients by 18% [7]. Since CPR is performed in a team, there is evidence suggesting that it affects NTS and CPR results [8]. A study showed a clear difference in leadership and task distribution between the team that succeeded in CPR and the team that failed [9]. Hunziker et al. found significant differences in the chest compression start time, the total chest compression time, and the chest compression speed when the technique training group and the leadership training group were compared [10]. 

Currently, there are no randomized controlled trials in which teamwork is evaluated in association with patient outcomes after a cardiac arrest [11]. Teamwork and leadership are recognized as important aspects of medicine [12]. Resuscitation efforts are delivered using complex, high-risk, and dynamic methods and require efficient interprofessional team coordination and collaboration to deliver safe and high-quality care. Leadership and teamwork training involves building a team of high-performing healthcare professionals from a group of highly skilled clinicians to provide efficient resuscitation to improve patient outcomes. The 2015 European Resuscitation Council guidelines highlighted that NTS training, including training in teamwork, leadership, and structured communication skills, are essential adjuncts to skills training, especially for those expected to perform cardiopulmonary resuscitation (CPR) [13].

Paramedics are responsible for initiating advanced life support during an out-of-hospital cardiac arrest (OHCA), which is a vital link in the chain of survival. Prehospital resuscitation is more difficult than in-hospital resuscitation. During OHCA, personnel with varying degrees of technical skills and NTSs form an improvised team to participate in CPR. In addition, stress, fatigue, and the frequent switching of positions are common factors that may threaten the teamwork of paramedics [14]. Further, the prehospital resuscitation procedure lacks human and material resources, is prone to litigation, and is often criticized by medical institutions and guardians, which puts the paramedics in a mentally vulnerable state [15]. Therefore, strong leadership and teamwork skills are needed, especially during prehospital resuscitations [16].

The standard measures of prehospital team performance are lacking, which makes it difficult to quantitatively assess the team performance of emergency medical services (EMS) [17]. This may be because, in critical, real-life situations, there are not enough resources to treat patients; hence, there are no experts to participate in the objective evaluation. As a result, most prehospital research is based on simulation training [11].

Therefore, this study aimed to evaluate the TEAM [18], which is considered to be the most promising approach in CPR situations among the currently known teamwork evaluation tools [19], to prehospital situations, and confirm its suitability in prehospital CPR performed by the EMS.

## 2. Materials and Methods

### 2.1. Patients Selection 

This was a multi-centric observational study. Teamwork was evaluated by experts using the video data recorded from the Smart Advanced Life Support (SALS) registry. SALS is a method of advanced resuscitation performed by paramedics under a video communication-based direction for patients older than 18 years with OHCA of non-traumatic causes in South Korea [20]. After arriving at the field, the paramedics used a mobile application to request medical direction from an emergency physician through video call, after which the on-site performance of the paramedics and the doctor’s medical guidance were recorded. If using the application was not possible, the medical direction was provided via video or voice calls.

### 2.2. Intervention

From 1 January 2018 to 31 December 2018, among the 2536 cases that were recorded in the SALS registry, 1725 cases for which the application was used were selected. Further, 1339 of the selected cases were extracted by excluding the cases in which it was difficult to evaluate teamwork due to poor audio and video conditions and short on-site CPR time (<10 min). To ensure the representativeness of the sample, 5% of the population was selected through simple randomization [21]. Finally, 67 cases were analyzed.

**Figure 1:**
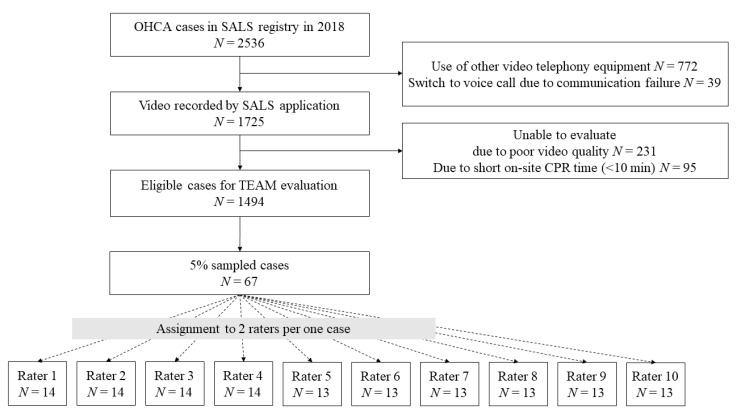
Flowchart of selection of eligible cases and allocation to raters. Abbreviations: OHCA, out-of-hospital cardiac arrest; SALS, Smart Advanced Life Support; CPR, cardiopulmonary resuscitation; TEAM, Team Emergency Assessment Measure.

Ten experts participated in the video evaluation. The expert group consisted of emergency physicians who conducted direct medical directions. The median age was 41 years, and males accounted for 81.8% of all the experts. They had 15 years of experience as an emergency physician and 48 months as medical directors for SALS (Table 1). Two experts were assigned to evaluate the teamwork for each case according to a predetermined sequence. Each expert reviewed 13 or 14 videos and evaluated the prehospital teamwork of each team (Figure 1).

### 2.3. Instrument

The TEAM developed by Cooper et al. was used as a tool for prehospital teamwork evaluation [18]. It consists of 11 items in 3 categories: leadership, teamwork, and task management. These items were rated on a 5-point Likert scale (‘0’ = never to ‘4’ = always) with a total score ranging from 0 to 44. The overall performance was rated on a global rating scale, ranging from 1 to 10. The original English version of the TEAM was translated into Korean according to World Health Organization guidelines [22]. Following forward translation, expert-panel back-translation, pretesting, and cognitive interviewing, the final version was completed, which was approved for use by the developer.

The raters participating in this study were emergency physicians directing medical interventions through video and consisted of advanced life support training managers for paramedics. The training course is based on a scenario in which a video-based medical direction is received during resuscitation. Therefore, the selected raters thoroughly understood the gap between the video performance and the actual situation. All the raters completed a 30-minute theoretical tutorial on the TEAM before evaluation and agreed upon the scoring level through trial evaluations of three sample cases. The evaluations were conducted in separate spaces to prevent the raters from sharing their results with each other. 

### 2.4. Ethical Approval

This study was approved by the relevant Institutional Review Board of the Hallym University Dongtan Sacred Heart Hospital (approval number: HDT 2019-12-011). Written informed consent was obtained from all the participants of this study. 

### 2.5. Statistical Analysis

All analyses were performed using SPSS v. 25 (IBM, New York, NY, USA). The baseline participant and event characteristics of the OHCAs are reported as numbers and percentages for qualitative variables and median and interquartile ranges for continuous variables. The measurement properties of the TEAM were evaluated for internal consistency, concurrent validity, inter-rater reliability, and floor/ceiling effect.

The internal consistency was examined using the mean interitem and item–total correlations and Cronbach’s alpha coefficient. An item–total correlation of >0.40 and Cronbach’s alpha >0.70 were considered satisfactory [23]. The concurrent validity was evaluated through the correlation of item scores with global ratings. The inter-rater reliability was measured using the intraclass correlation coefficient (ICC) with a 95% confidence interval. An ICC value of less than 0.4 was regarded as poor agreement [24]. The floor effect includes the proportion of observations with the lowest possible scores, whereas the ceiling effect represents observations with the highest possible score. The floor and ceiling effects of less than 15% of the observations were considered adequate [23]. 

## 3. Results

### 3.1. Characteristics of Clinical Cases

Table 2 shows the characteristics of the cardiac arrest cases used for evaluation. The median age of the patients was 61 years. Men accounted for 65.7% of the patients, cardiac arrest was witnessed in 64.2% of the patients, and 31.3% of the cases had an initial shockable rhythm. The median scene time for prehospital management was 21 minutes, and 50.7% of the patients had a prehospital return of spontaneous circulation (ROSC).

### 3.2. Measurement Properties 

The TEAM showed high reliability with a Cronbach’s alpha value of 0.939, and Cronbach’s alpha values for each item of leadership, teamwork, and task management were 0.828, 0.913, and 0.895, respectively. The interitem correlation ranged from 0.463 to 0.811, and the mean interitem correlation was 0.584. The mean item–total correlation was 0.789 (*r* = 0.708–0.822), which indicates significant item–total score associations (Table 3).

The median score for the single-item global rating was 6 (IQR, 4–7), and the median TEAM was 24.50 (IQR, 17.75–30.25). The mean correlation coefficient between each item and the global score range was 0.682 (*r* = 0.595 to 0.741, all *p* < 0.001), indicating good concurrent validity. The mean intraclass correlation coefficient (ICC) was 0.804, with excellent agreement between the raters. However, in 1 of the 67 clinical events, the ICC was 0.323, indicating poor agreement.

**Figure 2:**
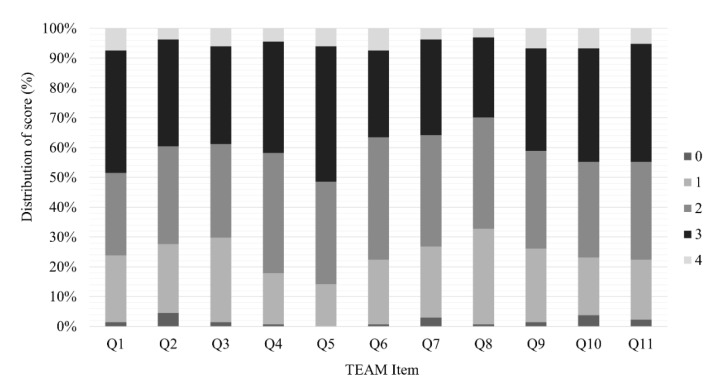
Distribution of the results of the TEAM. Abbreviation: TEAM, Team Emergency Assessment Measure.

The distribution of the lowest and highest scores was within 15% of all the items, which showed no floor or ceiling effects (Figure 2).

## 4. Discussion

To the best of our knowledge, this is the first study to investigate the validity of a tool that evaluates teamwork during a prehospital cardiac arrest situation rather than a simulation. In this study, the feasibility of the evaluation of teamwork was also suggested even in prehospital OHCA situations.

Although the importance of teamwork in the prehospital stage is sufficiently recognized, only a few studies were conducted due to the lack of data sources. This is because it is challenging to evaluate leadership and communication skills, which are the elements of NTS, only with paper records. Digital technology, such as video records, can be used as a reliable source for situational evaluation in the prehospital stage [25].

To determine the effectiveness of intervention programs to improve team performance, a valid and reliable tool is required. The Clinical Teamwork Scale [26], Observational Skill-based Clinical Assessment Tool for Resuscitation [27], Modified Non-Technical Skills Scale for Trauma [28], and TEAM [18] are well-known tools applicable to critical situations. Among these tools, the TEAM has shown validity in clinical settings and among staff and trainees. It is considered the most promising tool given its measurement of evidence [19]. However, no teamwork evaluation tool, including the TEAM, has been verified in actual prehospital clinical settings. Therefore, this study evaluated whether the TEAM is the most reliable measurement that can be used in prehospital CPR situations.

The mean score for each item in the TEAM was 1.99–2.43, which was lower than that of a previous study (2.69–3.30) in the in-hospital situation. The mean total score was 24.26, which was very different from 34.65 in that previous study [28]. The scores of the leadership category were lower in the in-hospital resuscitation situation [28], but the scores for responding to a changing situation and re-evaluating the situation were low during prehospital resuscitation. These results reflect the characteristics of prehospital situations. Compared with the in-hospital cardiac arrest patients, the OHCA patients were remarkably similar in demographics and most comorbidities, but they had low ROSC and survival rates [29]. In Korea, when a cardiac arrest occurs, a two-tiered EMS system is operated, i.e., two teams are immediately merged to form one team. The EMS that arrives first must respond in an early stage with two or three paramedics. However, during an unrecognized cardiac arrest, the activation of the second EMS team is delayed, making it more difficult to deal with manpower [15]. In addition, there is a lack of facilities and equipment, and it is also affected by the weather and location of the patient. Insufficient human and material resources and several unpredictable factors impede situational adaptation and assessment. 

In our study, the internal consistency of prehospital resuscitations was high, with a Cronbach’s alpha value of 0.939, which was similar to that in the previous study (Cronbach’s alpha = 0.94) that evaluated in-hospital resuscitation situations [30]. In addition, the mean interitem and item–total correlations in our study were strongly significant at 0.584 and 0.789, respectively, which were similar to those of the in-hospital resuscitations (mean interitem correlation, (*r* = 0.60); mean item–total correlation (*r* = 0.795)) [30]. 

In the concurrent validity evaluation, the item ratings were significantly associated with the global ratings (*r* = 0.682); however, this correlation was low compared with that of the evaluation conducted using video records of simulations (*r* = 0.80 to 0.94) [31] and the French version of the simulation study (*r* = 0.78) [32]. The mean ICC of the 11 TEAM items showed a high agreement rate of 0.804, with the exception of one case that showed poor agreement. The video of this case was reviewed by the authors, and it was observed that the performance of the paramedics was very poor; therefore, both raters gave 0 or 1 point for all the items. The ICC should be interpreted with caution, as it is affected by the range of measurements in the study population. For example, if the measured values of the study group were all high, and the range was small, the interindividual variation would be smaller than the intraindividual variation, and the ICC would be low.

This study has several limitations. First, we did not conduct evaluations in real time during the resuscitation. We analyzed and evaluated video records. Moreover, it was difficult to evaluate the leader’s situational awareness, decision-making ability, or skills by simply observing their behavior. To reduce the error caused by this, only the expert group of video-guided medical directors were designated as the raters in this study. Second, we could not calculate the test–retest reliability. The videos used for analysis were stored on a secure server to protect patient privacy and could only be reviewed on a designated computer after prior approval. Due to this, it was difficult to perform retesting. Hence, for the various case reviews in this study, two raters were assigned to each case to calculate the inter-rater reliability. Third, because the video data could not be processed, the raters could not check whether the patient had an on-site ROSC. It is possible that the patient’s outcome, along with the performance of the paramedics, may have affected the TEAM score. 

## 5. Conclusions

NTSs in the prehospital stage should be appropriately evaluated for patient safety. The TEAM can be a valid and reliable tool to evaluate the efficiency of a team of healthcare workers during real-life CPR situations.

## Figures and Tables

**Table 1:** Demographic characteristics of experts (*n* = 10).

Characteristics	*n* (%) or Median (IQR)
Age	41 (38–43)
Sex, male	9 (81.8)
Career of clinical physician (years)	15 (12–18)
Career of medical director by videophone (months)	48 (24–61)

IQR, interquartile range.

**Table 2:** Characteristics of patients and events (*n* = 67).

Characteristics	*n* (%)
Age, years, median (IQR)	61 (50–71)
Sex, male	44 (65.7)
Witnessed	43 (64.2)
Initial rhythm	
Shockable	21 (31.3)
Non-shockable	46 (68.7)
Scene time interval, min	21 (19–28)
Prehospital ROSC	34 (50.7)

IQR, interquartile range; ROSC, return of spontaneous circulation.

**Table 3:** The TEAM outcomes (67 clinical cases, 134 observations).

	Median (IQR)	Score Range *	Item–Total Correlation	*p*-Value
Q1. The team leader let the team know what was expected of them through direction and command.	2 (2–3)	0–4	0.759	<0.001
Q2. The team leader maintained a global perspective	2 (1–3)	0–4	0.807	<0.001
Q3. The team communicated effectively.	2 (1–3)	0–4	0.793	<0.001
Q4. The team worked together to complete tasks in a timely manner.	2 (2–3)	0–4	0.765	<0.001
Q5. The team acted with composure and control.	3 (2–3)	1–4	0.708	<0.001
Q6. The team morale was positive.	2 (2–3)	1–4	0.800	<0.001
Q7. The team adapted to changing situations.	2 (1–3)	0–4	0.814	<0.001
Q8. The team monitored and reassessed the situation.	2 (1–3)	0–4	0.797	<0.001
Q9. The team anticipated potential situations.	2 (1–3)	0–4	0.822	<0.001
Q10. The team prioritized tasks.	2 (2–3)	0–4	0.806	<0.001
Q11. The team followed approved standards/guidelines	2 (2–3)	0–4	0.803	<0.001
Total mean.	25 (18–30)			

TEAM, Team Emergency Assessment Measure; SD, standard deviation. * Score range: 0 = never or hardly ever; 1 = seldom; 2 = about as often as not; 3 = very often; 4 = always/nearly always.

## Data Availability

The data used to support the findings of this study are available from the corresponding author upon request. The original video data used in this study cannot be disclosed to protect personal information.
